# Renal Cell Carcinoma with bone metastases isn’t always bad

**DOI:** 10.18632/oncotarget.27076

**Published:** 2019-07-16

**Authors:** Fiorella Ruatta, Laurence Albiges, Lisa Derosa

**Affiliations:** Department of Cancer Medicine, Gustave Roussy Cancer Campus, Paris-Sud University, Villejuif, France

**Keywords:** bone metastases, RCC, synchronous solitary bone metastasis, radical resection, prognostic factors

The last several years have seen dramatic clinical advances in the diagnosis and treatment of metastatic renal cell carcinoma (mRCC). Most of this success has been due to tyrosine kinase inhibitors (TKI) and T cell checkpoint inhibitors, agents which have revolutionized the treatment of some cancers, including the RCC.

As a consequence, approximately 20–30% of patients with mRCC developed bone metastases (BMs) [1] with a probability that was parallel to greater survival related to the introduction of new therapies [2]. Unfortunately, in presence of BMs the expected survival is poor, and in most cases, only palliative care is indicated. Generally, this conduct seems to be justified. BMs are mainly osteolytic and lead to skeletal-related events (SREs), including pathologic fractures, spinal cord compression and hypercalcemia, all associated with an impairment of quality of life [3]. Specifically, BMs have been identified as an independent prognostic variable predicting poor overall survival (OS) irrespective of prognostic factors by the International Metastatic Renal Cell Carcinoma Database Consortium (time from diagnosis to systemic therapy, Karnofsky Performance Status, levels of hemoglobin, corrected calcium, neutrophils and platelets) [4]. In addition, the International Kidney Cancer Working Group identified BMs treated with TKI as conferring significantly poorer OS than other metastatic sites but equally to hepatic metastases [5]. Nevertheless, median OS after diagnosis of RCC BM’s ranges from 12 to 40 months [1, 6] and subgroups of patients surviving longer have been identified.

This optimistic view is supported by our and other works which found out prognostic features associated with BMs. In the Kume cohort analyzing 94 patients with BMs from RCC [7], 5 factors are defined as right associated with the unfortunate prognosis: sarcomatoid differentiation, spinal involvement, extraosseous metastasis, increased alkaline phosphatase and C-reactive protein levels. Similarly, Santoni *et al.* determined patients’ age, ECOG performance status, histology, Memorial Sloan Kettering Cancer Center (MSKCC) score, time to BMs and the presence of concomitant metastases to be significant related with poor OS [6].

In order to identify patients with the less-aggressive BMs from RCC, we analyzed 1750 patients with a diagnosis of RCC and treated at Gustave Roussy, estimating 300 (17%) patients with BMs [1]. Risk factors associated with a poorer prognosis were three: MSKCC risk group, presence of more than 5 BMs, and other sites of disease. Inversely, patients presenting with a single solitary bone lesion (SSBM) at the diagnosis of metastatic disease had longer OS (40 vs. 20 months, *p* < 0.001). Although this represented a small subgroup (7%) of patients, the increased likelihood to having undergone radical resection could explain this prognosis. Indeed, independently from other factors, radical surgical resection of BMs was associated with improved survival whereas palliative surgery, radiotherapy and other local or systemic treatments were not. Decisively in our work, a patient with diagnosis of BMs and favorable prognosis was characterized by a SSBM subjected to radical resection, good MSKCC score and no visceral metastases. In short, the presence or absence of metastases, including BMs, is crucial for the choice of treatment as advised by a recent Expert Consensus regarding whether wide resection with curative intent in solitary or oligometastatic BMs patients is recommended [2]. The role of other local treatment of metastases such as radiotherapy, cryotherapy, radiofrequency remains controversial. For these reasons, we strongly support a multidisciplinary team approach in order to personalize treatment planning and prolong survival of these patients. Finally, we encourage a better understanding of the mechanistic links involved in regulation of mRCC, immune system and skeletal metastases. Several pathways are involved including transforming growth factor-beta (TGF-beta), bone morphogenic proteins (BMPs), bone sialoproteins (BSP), calcium-sensing receptor (CaSR), insuline – like growth factor (IGF) mRNA binding protein-3, cadherin-11, AKT/integrin-5 signaling system, parathyroid hormone-related protein (PTHrP) and matriptase [6] but still we are in need of further improvement in the pathophysiology of RCC metastatic spread to bone. This will allow the optimal design of therapies for BMs in RCC patients.

## References

[1] Ruatta F, et al Eur J Cancer. 2019; 107:79–85. 10.1016/j.ejca.2018.10.023. 30551078

[2] Grünwald V, et al Nat Rev Urol. 2018; 15:511–21. 10.1038/s41585-018-0034-9. 29904105 PMC7136176

[3] Woodward E, et al Bone. 2011; 48:160–66. 10.1016/j.bone.2010.09.008. 20854942

[4] McKay RR, et al Eur Urol. 2014; 66:502–09. 10.1016/j.eururo.2014.02.040. 24613250 PMC4145043

[5] McKay RR, et al Eur Urol. 2014; 65:577–84. 10.1016/j.eururo.2013.08.012. 23962746 PMC4123121

[6] Santoni M, et al J Exp Clin Cancer Res. 2015; 34:10. 10.1186/s13046-015-0122-0. 25651794 PMC4328067

[7] Kume H, et al J Urol. 2011; 185:1611–14. 10.1016/j.juro.2010.12.037. 21419440

